# Supplementary material for: Modeling glioblastoma heterogeneity as a dynamic network of cell states
Source: Mol Syst Biol. 2021 Sep 16;17(9):e10105. doi: 10.15252/msb.202010105 (PMC8444284; doi:10.15252/msb.202010105)
Supplement: Supplementary file 6 — Source Data for Figure 5 [file MSB-17-e10105-s004.zip › Figure5A_sourcedata/GSEA_3071/hallmarks_stateIII.GseaPreranked.1623742540963/HALLMARK_UNFOLDED_PROTEIN_RESPONSE.html]

Details for gene set HALLMARK\_UNFOLDED\_PROTEIN\_RESPONSE[GSEA]

|  || Dataset | state33071 |
| Phenotype | NoPhenotypeAvailable |
| Upregulated in class | na\_pos |
| GeneSet | HALLMARK\_UNFOLDED\_PROTEIN\_RESPONSE |
| Enrichment Score (ES) | 0.15102008 |
| Normalized Enrichment Score (NES) | 1.0065557 |
| Nominal p-value | 0.47244096 |
| FDR q-value | 0.4373698 |
| FWER p-Value | 0.857 |
Table: GSEA Results Summary

  

Fig 1: Enrichment plot: HALLMARK\_UNFOLDED\_PROTEIN\_RESPONSE      
 Profile of the Running ES Score & Positions of GeneSet Members on the Rank Ordered List

  

| PROBE | GENE SYMBOL | GENE\_TITLE | RANK IN GENE LIST | RANK METRIC SCORE | RUNNING ES | CORE ENRICHMENT || 1 | NPM1 |  |  | 88 | 0.421 | 0.0191 | Yes |
| 2 | EIF4A1 |  |  | 150 | 0.374 | 0.0404 | Yes |
| 3 | RPS14 |  |  | 196 | 0.340 | 0.0625 | Yes |
| 4 | NHP2 |  |  | 232 | 0.322 | 0.0854 | Yes |
| 5 | LSM4 |  |  | 268 | 0.303 | 0.1064 | Yes |
| 6 | EIF4EBP1 |  |  | 316 | 0.287 | 0.1227 | Yes |
| 7 | BANF1 |  |  | 317 | 0.287 | 0.1510 | Yes |
| 8 | DNAJB9 |  |  | 448 | -0.251 | 0.1425 | No |
| 9 | ATF3 |  |  | 515 | -0.255 | 0.1508 | No |
| 10 | PDIA5 |  |  | 715 | -0.265 | 0.1260 | No |
| 11 | DNAJC3 |  |  | 780 | -0.268 | 0.1361 | No |
| 12 | TATDN2 |  |  | 833 | -0.271 | 0.1496 | No |
| 13 | SSR1 |  |  | 993 | -0.279 | 0.1364 | No |
| 14 | CALR |  |  | 1498 | -0.306 | 0.0375 | No |
| 15 | GEMIN4 |  |  | 1648 | -0.315 | 0.0304 | No |
| 16 | ERN1 |  |  | 1657 | -0.316 | 0.0596 | No |
| 17 | FKBP14 |  |  | 1860 | -0.327 | 0.0402 | No |
| 18 | DDIT4 |  |  | 2027 | -0.337 | 0.0309 | No |
| 19 | XBP1 |  |  | 2097 | -0.341 | 0.0470 | No |
| 20 | WFS1 |  |  | 2307 | -0.358 | 0.0287 | No |
| 21 | NFYA |  |  | 2324 | -0.360 | 0.0602 | No |
| 22 | EDEM1 |  |  | 2411 | -0.366 | 0.0743 | No |
| 23 | PDIA6 |  |  | 2505 | -0.373 | 0.0874 | No |
| 24 | STC2 |  |  | 2570 | -0.379 | 0.1085 | No |
| 25 | EIF2AK3 |  |  | 2620 | -0.384 | 0.1338 | No |
| 26 | SLC1A4 |  |  | 3051 | -0.426 | 0.0657 | No |
| 27 | HSPA5 |  |  | 3193 | -0.442 | 0.0732 | No |
| 28 | NABP1 |  |  | 3405 | -0.481 | 0.0667 | No |
| 29 | HSP90B1 |  |  | 3757 | -0.681 | 0.0441 | No |
Table: GSEA details [plain text format]

  

Fig 2: HALLMARK\_UNFOLDED\_PROTEIN\_RESPONSE: Random ES distribution      
 Gene set null distribution of ES for **HALLMARK\_UNFOLDED\_PROTEIN\_RESPONSE**
